# Prognostic impact of adjuvant endocrine therapy for estrogen receptor-positive and HER2-negative T1a/bN0M0 breast cancer

**DOI:** 10.1007/s10549-023-07097-6

**Published:** 2023-09-09

**Authors:** Shinsuke Sasada, Naoto Kondo, Hiroya Hashimoto, Yuko Takahashi, Kaori Terata, Kumiko Kida, Yasuaki Sagara, Takayuki Ueno, Keisei Anan, Akihiko Suto, Chizuko Kanbayashi, Mina Takahashi, Rikiya Nakamura, Toshiyuki Ishiba, Michiko Tsuneizumi, Seiichiro Nishimura, Yoichi Naito, Fumikata Hara, Tadahiko Shien, Hiroji Iwata

**Affiliations:** 1 Department of Surgical Oncology, Research Institute for Radiation Biology and Medicine, Hiroshima University, Hiroshima, Japan; 2 Department of Breast Surgery, Nagoya City University Graduate School of Medical Sciences, Nagoya, Japan; 3 Core Laboratory, Nagoya City University Graduate School of Medical Sciences, Nagoya, Japan; 4 Department of Breast and Endocrine Surgery, Okayama University Hospital, 2-5-1 Shikata-cho, Kitaku, 700-8558 Okayama Japan; 5 Department of Breast and Endocrine Surgery, Akita University Hospital, Akita, Japan; 6 Department of Breast Surgical Oncology, St. Luke’s International Hospital, Tokyo, Japan; 7 Department of Breast and Thyroid Surgical Oncology, Social medical corporation Hakuaikai, Sagara Hospital, Kagoshima, Japan; 8 Breast Oncology Center, The Cancer Institute Hospital of Japanese Foundation for Cancer Research, Tokyo, Japan; 9 Department of Surgery, Kitakyushu Municipal Medical Center, Kitakyushu, Japan; 10 Department of Breast Surgery, National Cancer Center Hospital, Tokyo, Japan; 11 Department of Breast Oncology, Niigata Cancer Center Hospital, Niigata, Japan; 12 Department of Breast Oncology, National Hospital Organization Shikoku Cancer Center, Matsuyama, Japan; 13 Department of Breast Surgery, Chiba Cancer Center, Chiba, Japan; 14 Department of Breast Surgery, Tokyo Metropolitan Cancer and Infectious Diseases Center, Komagome Hospital, Tokyo, Japan; 15 Department of Breast Surgery, Shizuoka General Hospital, Shizuoka, Japan; 16 Department of Breast Surgery, Shizuoka Cancer Center Hospital, Shizuoka, Japan; 17 Department of General Internal Medicine, National Cancer Center Hospital East, Chiba, Japan; 18 Department of Breast Oncology, Aichi Cancer Center Hospital, Nagoya, Japan

**Keywords:** Breast cancer, T1a/b, Endocrine therapy, Estrogen receptor, Prognosis

## Abstract

**Purpose:**

Mammography screening has increased the detection of subcentimeter breast cancers. The prognosis for estrogen receptor (ER)-positive and human epidermal growth factor receptor 2 (HER2)-negative T1a/bN0M0 breast cancers is excellent; however, the necessity of adjuvant endocrine therapy (ET) is uncertain.

**Methods:**

We evaluated the effectiveness of adjuvant ET in patients with ER-positive and HER2-negative T1a/bN0M0 breast cancer who underwent surgery from 2008 to 2012. Standard ET was administrated after surgery. The primary endpoint was the cumulative incidence of distant metastasis. All statistical tests were 2-sided.

**Results:**

Adjuvant ET was administered to 3991 (83%) of the 4758 eligible patients (1202 T1a [25.3%] and 3556 T1b [74.7%], diseases). The median follow-up period was 9.2 years. The 9-year cumulative incidence of distant metastasis was 1.5% with ET and 2.6% without ET (adjusted subdistribution hazard ratio [sHR], 0.54; 95% CI, 0.32–0.93). In multivariate analysis, the independent risk factors for distant metastasis were no history of ET, mastectomy, high-grade, and lymphatic invasion. The 9-year overall survival was 97.0% and 94.4% with and without ET, respectively (adjusted HR, 0.57; 95% CI, 0.39–0.83). In addition, adjuvant ET reduced the incidence of ipsilateral and contralateral breast cancer (9-year rates; 1.1% vs. 6.9%; sHR, 0.17, and 1.9% vs. 5.2%; sHR, 0.33).

**Conclusions:**

The prognosis was favorable in patients with ER-positive and HER2-negative T1a/bN0M0 breast cancer. Furthermore, adjuvant ET reduced the incidence of distant metastasis with minimal absolute risk difference. These findings support considering the omission of adjuvant ET, especially for patients with low-grade and no lymphatic invasion disease.

**Supplementary Information:**

The online version contains supplementary material available at 10.1007/s10549-023-07097-6.

## Introduction

Breast cancer is the most common cancer worldwide, affecting 2.3 million people annually and accounting for approximately a quarter of all female cancers [1, 2]. Approximately 70% of patients have cancers that are estrogen receptor (ER)-positive and human epidermal growth factor receptor 2 (HER2)-negative [3]. Notably, screening mammography has increased the detection of small breast cancers, with an approximately 5-fold increase in the incidence of subcentimeter breast cancers (from 12.8 to 66.0 per 100,000 women) [4]. In Japan, approximately 19,000 patients with T1a/b breast cancer annually undergo surgery [5]. Patients with T1a/b breast cancer, almost N0, have an excellent prognosis, with a 10-year breast cancer mortality rate of 3.2–4% [4, 6] and a 10-year recurrent-free survival of over 90% [7]. These clinical outcomes were comparable to those of distant metastasis-free intervals (DMFIs) in particularly low-risk cases using multi-gene assays, such as the 9-year DMFI 96.8% in cases with a recurrence score of ≤ 10 using a 21-gene assay and the 8-year DMFI 97.0% in ultralow-risk cases using the 70-gene signature [8, 9].

Adjuvant endocrine therapy (ET) is a global standard treatment for ER-positive breast cancer, including T1a/b disease [10–13]. The national comprehensive cancer network (NCCN) guideline classifies adjuvant ET for T1aN0 breast cancer as category 2B, meaning that “based upon lower-level evidence, the NCCN consensus prescribes that the intervention is appropriate.” The national surgical adjuvant breast and bowel project (NSABP)-B21 trial for T1a/bN0 breast cancer demonstrated that the distant metastasis rates were 3.3% in the radiotherapy + placebo group and 1.6% in the radiotherapy + tamoxifen group (*P* = .28) [14]. However, a previous cohort study of T1 breast cancer reported that the 7-year cumulative incidence of metastasis was 2%, and ET was not a predictor [15]. Therefore, the necessity of adjuvant ET for T1a/bN0 breast cancer remains uncertain.

Several patients discontinue adjuvant ET despite the recommendations of clinical guidelines. For instance, 31–73% of patients discontinued ET at five years of treatment, with a mean adherence rate of 66.2% [16, 17]. Notably, side effects of ET, such as hot flashes, arthralgia, fatigue, mood disturbance, and vaginal bleeding, cause long-term patient distress [18]. Additionally, ET is reported to persistently deteriorate the quality of life (QOL) [19]. Therefore, the selection of patients for adjuvant ET is necessary.

Furthermore, the patient involvement committee of the Japan clinical oncology group (JCOG) Breast Cancer Study Group discussed treatment preferences with patients, and one of the requests from patient advocators was to omit adjuvant ET if the clinical effect was minimal. In Japan, 86.3% of patients with ER-positive and HER2-negative stage I breast cancer underwent adjuvant ET in 2018 [5]. Therefore, we conducted a multicenter cohort study to investigate the long-term prognosis and effect of adjuvant ET in ER-positive and HER2-negative T1a/bN0M0 breast cancer.

## Materials and methods

### Study population

We retrospectively collected the medical data of patients with ER-positive and HER2-negative T1a/bN0M0 breast cancer who underwent radical surgery from January 2008–December 2012 in 42 JCOG Breast Cancer Study Group institutes. Tumor size and negative nodes were pathologically defined, and microinvasive disease (T1mi) was included in T1a. ER positivity was defined as ≥ 1% staining by immunohistochemistry [20]. We excluded patients who received neoadjuvant systemic therapy and those with confirmed pathogenic *BRCA* variants.

### Statistical analysis

Baseline characteristics are described as numbers and percentages. Fisher’s exact test was used to compare the frequencies of the categorical variables. The primary endpoint was the cumulative incidence of distant metastasis. The secondary endpoints were distant disease-free survival (DDFS), disease-free survival (DFS), overall survival (OS), ipsilateral breast tumor recurrence (IBTR), and contralateral breast cancer. The effect of adjuvant ET was analyzed using Gray’s test model for cumulative incidence and log-rank test for survival. Predictive factors were assessed using the Fine-Gray model and Cox proportional hazards model. Hazard ratios (HRs) were adjusted by age, tumor size, nuclear grade, Ki-67 labeling index, lymphovascular invasion, and treatment. Statistical significance was defined as a two-sided *P* value < 0.05. All statistical analyses were performed using the SAS statistical software version 9.4 (SAS Institute).

The consensus of the investigators was that adjuvant ET would be the standard of care if the absolute difference in distant metastasis was 3% or greater, and no ET would be the standard of care if the absolute difference was 1% or less. If the difference was between 1 and 3%, a prospective trial would be planned or considered for shared decision-making.

## Results

Among the 4914 patients with ER-positive and HER2-negative T1a/bN0M0 breast cancer who underwent surgery from January 2008–December 2012, 4758 were eligible for inclusion in this study (supplementary figure S1). Patient characteristics are presented in Table 1. Half were over 55 years old, 1202 (25.3%) had T1a tumors, and 3450 (72.5%) had undergone breast-conserving surgery. Adjuvant ET was administered to 3991 (83.9%) patients, and luteinizing hormone-releasing hormone (LH-RH) analog to 577 (34.7%) of 1661 premenopausal and 7 (13.0%) of 54 perimenopausal women. Absence of ET history was related to T1a tumor, low Ki-67 labeling index, mastectomy, and absence of radiation therapy history. The reasons for not performing ET were institutional policy and physician’s decision 395 (51.5%), patient preference 200 (26.1%), and co-morbidity 19 (2.5%). The median duration of ET was five years (ranging from 0 to 15). The rate of sufficient adherence, defined as the completion of 4.5 years of ET, was 83.9%. The median follow-up period was 9.2 years.

**Table 1:** Characteristics of Patients With T1a/b Breast Cancer

Variable	No. (%)			
	All	ET group	Non-ET group	
	(n = 4758)	(n = 3991)	(n = 767)	*P* value^a^
Gender				
Female	4753 (99.9)	3986 (99.9)	767 (100)	1
Male	5 (0.1)	5 (0.1)	0 (0)	
Age, y				
<55	2344 (49.3)	1969 (49.4)	375 (48.9)	0.813
≥55	2412 (50.7)	2020 (50.6)	392 (51.1)	
Menopausal status				
Premenopausal	1994 (42)	1661 (41.7)	333 (43.4)	0.042
Perimenopausal	57 (1.2)	54 (1.4)	3 (0.4)	
Postmenopausal	2637 (55.5)	2222 (55.7)	415 (54.1)	
Unknown	65 (1.4)	49 (1.2)	16 (2.1)	
Histology				
Infiltrating duct carcinoma NOS	4379 (92.1)	3680 (92.2)	699 (91.1)	0.550
Invasive lobular carcinoma	139 (2.9)	113 (2.8)	26 (3.4)	
Mucinous adenocarcinoma	137 (2.9)	114 (2.9)	23 (3.0)	
Others	103 (2.1)	84 (2.1)	19 (2.5)	
Tumor size, mm				
≤5	1202 (25.3)	835 (20.9)	367 (47.8)	< 0.001
>5	3556 (74.7)	3156 (79.1)	400 (52.2)	
Nuclear grade				
1	3052 (64.1)	2610 (65.4)	442 (57.6)	< 0.001
2	1105 (23.2)	865 (21.7)	240 (31.3)	
3	281 (5.9)	261 (6.5)	20 (2.6)	
Unknown	320 (6.7)	255 (6.4)	65 (8.5)	
Ki-67 labeling index, %				
0–9	918 (19.9)	748 (19.2)	170 (24.3)	0.001
10–19	660 (14.3)	564 (14.4)	96 (13.7)	
20–29	227 (4.9)	186 (4.8)	41 (5.8)	
30–39	109 (2.4)	92 (2.4)	17 (2.4)	
40–100	92 (2.0)	78 (2.0)	14 (2.0)	
Unknown	2599 (56.4)	2236 (57.3)	363 (51.8)	
Lymphatic invasion				
No	3999 (84.0)	3374 (84.5)	625 (81.5)	< 0.001
Yes	460 (9.7)	419 (10.5)	41 (5.3)	
Unknown	299 (6.3)	198 (5.0)	101 (13.2)	
Vascular invasion				
No	4325 (90.9%)	3667 (91.9%)	658 (85.8%)	< 0.001
Yes	114 (2.4)	109 (2.7)	5 (0.7)	
Unknown	319 (6.7)	215 (5.4)	104 (13.6)	
Progesterone receptor				
Positive	3448 (72.5)	2917 (73.1)	531 (69.2)	0.004
Negative	437 (9.2)	374 (9.4)	63 (8.2)	
Unknown	873 (18.3)	700 (17.5)	173 (22.6)	
Surgical procedure				
Breast-conserving surgery	3450 (72.5)	2975 (74.5)	475 (61.9)	< 0.001
Mastectomy	1153 (24.2)	888 (22.3)	265 (34.6)	
Skin-sparing mastectomy	88 (1.8)	68 (1.7)	20 (2.6)	
Nipple-sparing mastectomy	67 (1.4)	60 (1.5)	7 (0.9)	
Radiation therapy^b^				
No	501 (14.5)	347 (11.7)	154 (32.4)	< 0.001
Yes	2921 (84.7)	2610 (87.7)	311 (65.5)	
Unknown	28 (0.8)	18 (0.6)	10 (2.1)	
Endocrine therapy^c^				
Tamoxifen		2054 (51.5)	NA	-
Aromatase inhibitor		2142 (53.7)	NA	
LH-RH analog		595 (14.9)	NA	
Others		76 (1.9)	NA	

Abbreviations: ET, endocrine therapy; IQR, interquartile range; LH-RH, luteinizing hormone-releasing hormone; NA, not applicable; NOS, not otherwise specified

^a^*P* values estimated using the χ2 test

^b^ Includes only patients received breast conserving surgery

^c^ Includes duplicates

### Cumulative incidence of distant metastasis, DDFS, and OS

**Fig. 1:**
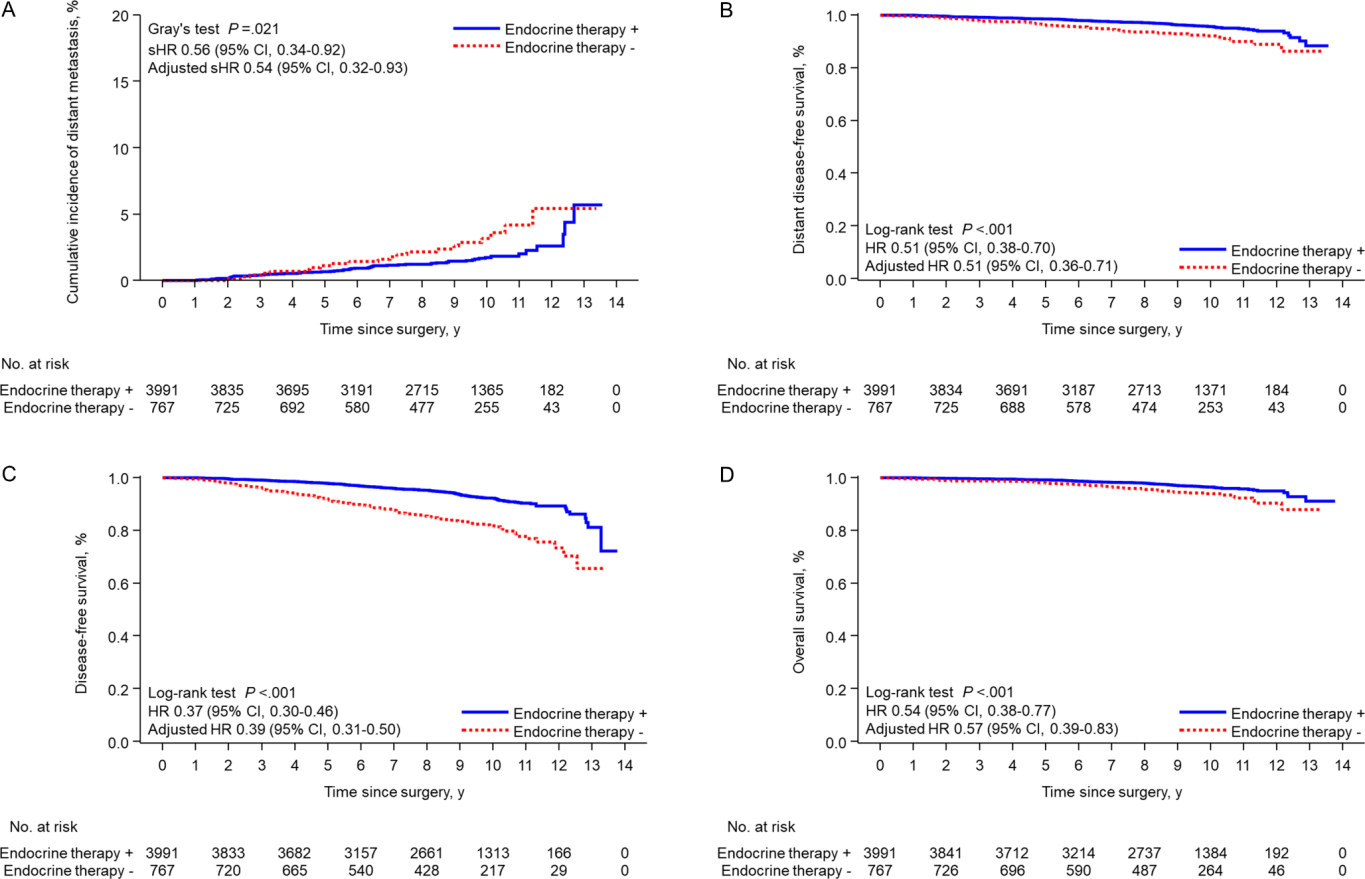
Disease outcomes were compared according to adjuvant endocrine therapy in patients with T1a/bN0M0 breast cancer. Cumulative incidence of distant metastasis (**A**), distant disease-free survival (**B**), disease-free survival (**C**), and overall survival (**D**)

Overall, 84 patients developed distant recurrence. The 9-year cumulative incidence of distant metastasis was 1.5% (95% confidence interval [CI], 1.1–1.9%) in the ET group and 2.6% (95% CI, 1.5–4.1%) in the non-ET group (adjusted subdistribution hazard ratio [sHR], 0.54; 95% CI, 0.32–0.93, *P* = .027) (Fig. 1A). The 9-year DDFSs were 96.2% (95% CI, 95.5–96.8%) and 92.9% (95% CI, 90.5–94.6%) in the ET and non-ET groups, respectively (adjusted HR, 0.51; 95% CI, 0.36–0.71, *P* < .001) (Fig. 1B). The 9-year DFSs were 93.6% (95% CI, 92.6–94.4%) and 83.5% (95% CI, 80.3–86.2%) in the ET and non-ET groups, respectively (adjusted HR, 0.39; 95% CI, 0.31–0.50, *P* < .001) (Fig. 1C). The 9-year OSs were 97.0% (95% CI, 96.3–97.5%) and 94.4% (95% CI, 92.2–96.0%), respectively (adjusted HR, 0.57; 95% CI, 0.39–0.83, *P* = .004 (Fig. 1D).

### Risk of distant metastasis and OS

In the multivariate analysis, risk factors for distant metastasis were high-grade, lymphatic invasion, mastectomy, and absence of ET history (Table 2). In contrast, the negative predictors of OS were older age, lymphatic invasion, and absence of ET history (supplementary table S1).

**Table 2:** Risk of Distant Metastasis in Patients With T1a/b Breast Cancer

Variable	Univariate analysis^a^		Multivariate analysis^a^	
	sHR (95% CI)	*P* value	sHR (95% CI)	*P* value
Age, y				
<55	Reference		Reference	
≥55	0.75 (0.48–1.16)	0.200	0.85 (0.55–1.31)	0.456
Tumor size, mm				
≤5	Reference		Reference	
>5	0.81 (0.50–1.30)	0.386	0.90 (0.53–1.54)	0.706
Nuclear grade				
1	Reference		Reference	
2	1.91 (1.15–3.18)	0.012	1.46 (0.84–2.56)	0.183
3	4.61 (2.58–8.24)	< 0.001	2.47 (1.01–6.03)	0.047
Ki-67 labeling index, %				
0–9	Reference		Reference	
10–19	5.51 (1.82–16.68)	0.003	1.75 (0.84–3.65)	0.138
20–29	3.13 (0.71–13.88)	0.133	1.20 (0.42–3.44)	0.737
30–39	10.56 (2.85–39.05)	< 0.001	2.19 (0.70–6.86)	0.177
40–100	11.56 (2.90–46.01)	< 0.001	2.47 (0.89–6.82)	0.082
Lymphatic invasion	2.81 (1.65–4.79)	< 0.001	2.32 (1.30–4.11)	0.004
Vascular invasion	0.52 (0.07–3.76)	0.517	0.46 (0.06–3.43)	0.449
Endocrine therapy	0.56 (0.34–0.92)	0.023	0.54 (0.32–0.93)	0.027
Surgical procedure				
Mastectomy	Reference			
Breast-conserving surgery	0.54 (0.35–0.85)	0.007	0.59 (0.37–0.93)	0.022
Radiation therapy	0.60 (0.39–0.93)	0.022	1.08 (0.51–2.28)	0.850

Abbreviations: CI, confidence interval; sHR, subdistribution hazard ratio

^a^ Estimated using the Fine-Gray models

### Distant metastasis according to risk factors

**Fig. 2:**
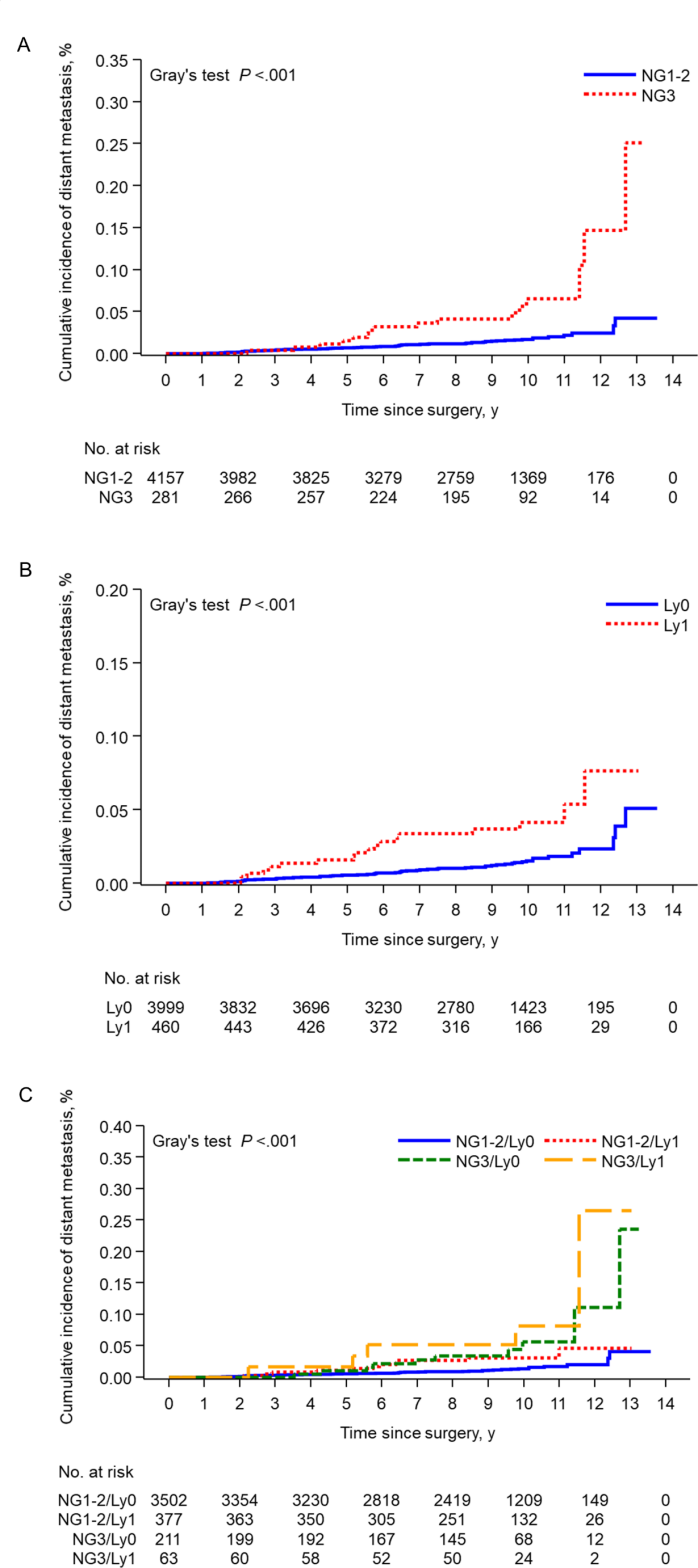
Cumulative incidence of distant metastasis according to risk factors. Nuclear grade (**A**), lymphatic invasion (**B**), and both (**C**). NG, nuclear grade; Ly, lymphatic invasion

The 9-year cumulative incidences of distant metastasis were 1.5% (95% CI, 1.1–1.9%) and 4.1% (95% CI, 2.1–7.2%) in cases of nuclear grade 1–2 and 3 (Gray’s test *P* < .001), and 1.2% (95% CI, 0.9–1.6%) and 3.7% (95% CI, 2.2–5.9%) in lymphatic invasion negative and positive cases (Gray’s test *P* < .001), respectively (Fig. 2A, B). Distant metastatic events were stratified by nuclear grade and lymphatic invasion (Gray’s test *P* < .001, Fig. 2C). The cumulative incidence of distant metastasis and the 9-year rates with and without ET for each risk group are described in supplementary figure S2 and table S2. The effect of ET was expected, even in the low-risk group. No difference in the 9-year cumulative incidence of distant metastasis according to　tumor size was observed (T1a: 1.7% and 2.7%; T1b: 1.4% and 2.5%, in the ET and non-ET groups, respectively) (supplementary figure S3).

### IBTR and contralateral breast cancer

**Fig. 3:**
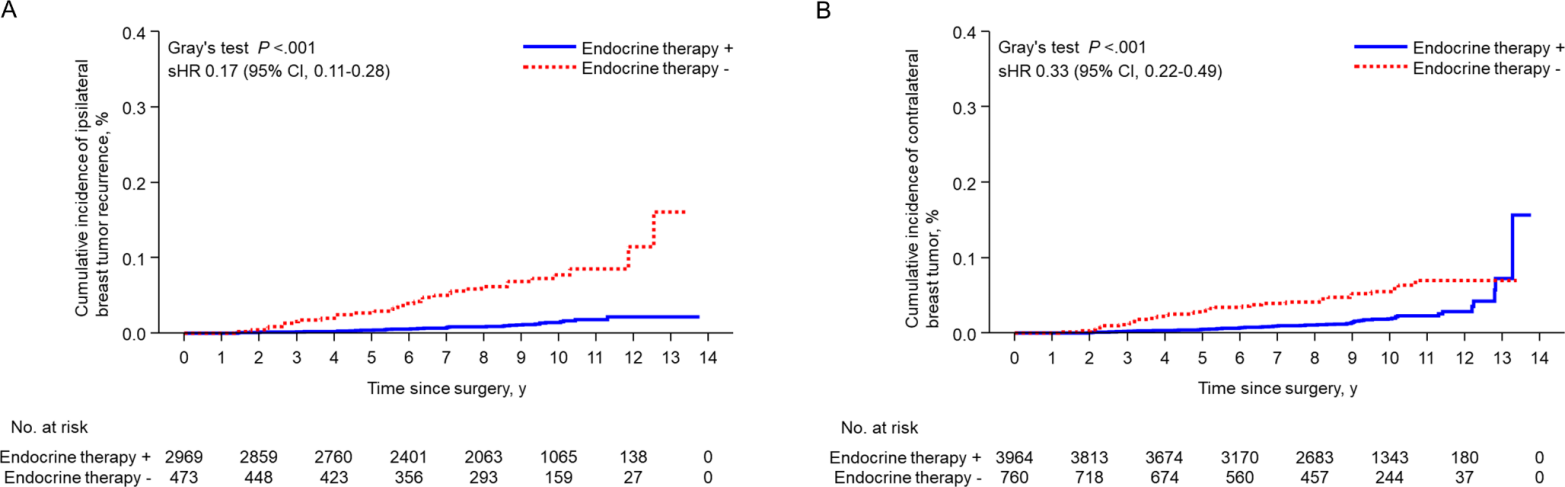
Cumulative incidence of ipsilateral (**A**) and contralateral (**B**) breast cancer according to adjuvant endocrine therapy in patients with T1a/bN0M0 breast cancer

IBTR was assessed only in patients who underwent breast-conserving surgery and included local recurrence and new primary tumors. The 9-year IBTRs were 1.1% (95% CI, 0.7–1.6%) in the ET group and 6.9% (95% CI, 4.6–9.7%) in the non-ET group (sHR, 0.17; 95% CI, 0.11–0.28, *P* < .001) (Fig. 3A). The risk factors for IBTR were younger age, high grade, vascular invasion, absence of ET history, and absence of radiation therapy history (supplementary table S3). The 9-year incidences of contralateral breast cancer were 1.4% (95% CI, 1.0–1.8%) and 5.2% (95% CI, 3.6–7.2%), respectively (sHR, 0.33; 95% CI, 0.22–0.49, *P* < .001) (Fig. 3B).

## Discussion

Among the 4758 patients with ER-positive and HER2-negative T1a/bN0M0 breast cancer, distant metastasis occurred in 84 patients. Adjuvant ET significantly reduced the cumulative incidence of distant metastasis by an absolute difference of 1.1% at 9 years after surgery. Pathologically, the nuclear grade and lymphatic invasion were independent risk factors for distant recurrence.

The prognosis of T1a/bN0M0 breast cancer was previously investigated; however, most studies included all subtypes [6, 7, 15, 21]. These findings consistently indicate that hormone receptor-negative and HER2-positive status are poor prognostic factors. Furthermore, the NSABP-B21 trial did not require ER status for registration, and approximately 80% of the known cases were ER-positive (HER2 status was not reported) [14]. At a median follow-up of 8 years, adding tamoxifen to radiation therapy after lumpectomy reduced distant recurrence by 1.8% in absolute value, without statistical significance.　Moreover, the integrated analysis for ER-positive T1a/bN0M0 breast cancer from three randomized studies (NSABP-B06, B-14, and B-20) revealed that adjuvant tamoxifen improved recurrence-free survival than surgery alone (HR, 0.55; 95% CI, 0.35–0.88) [22]. Furthermore, in a previous cohort study, the Early Breast Cancer Trialists’ Collaborative Group (EBCTCG) reported that 5-year adjuvant tamoxifen and aromatase inhibitors reduced recurrence risk for ER-positive breast cancer, including T1 disease (tamoxifen vs. control: rate ratio [RR], 0.60 and aromatase inhibitors vs. tamoxifen: RR 0.76) [23–25]. In addition, a previous cohort study of ER-positive and HER2-negative T1N0M0 breast cancer from a Japanese institute reported that adjuvant ET did not improve DFS, DDFS, or OS for T1a/b disease, unlike T1c [26]. However, the median follow-up period in this study was 60 months, which was relatively short. Distant recurrence of ER-positive breast cancer persisted beyond 5 years [27]. Here, the cumulative incidence of distant metastasis was 0.7% and 1.1% at 5 years, and 1.5% and 2.6% at 9 years in the ET and non-ET groups, respectively. The recurrence prevention effect of ET is maintained after the treatment period, and the long-term outcomes should be highlighted for ER-positive breast cancer [24, 28].

Furthermore, because the absolute risk reduction of distant metastasis in all cohorts was minimal, stratification by risk factors might be helpful for treatment decisions. Our findings and previous reports indicated tumor grade and lymphatic invasion as poor prognostic factors [6, 7, 15, 29]. The 9-year cumulative incidence of distant metastasis increased by approximately 2% for each additional risk factor. Unfortunately, almost all patients with two risk factors, especially high nuclear grade, underwent ET, and it was impossible to estimate the effect of ET for each risk factor.

One of the disadvantages of ET is its side effects, which include the following: hot flashes, arthralgia, fatigue, mood disturbance, and vaginal bleeding [18]. These symptoms afflict patients and worsen their QOL in the long term during the ET period [19, 30, 31]. Furthermore, several integrative therapies and medications have been examined, including meditation, relaxation, yoga, massage, and music therapy for mood disturbance; acupuncture, yoga, and duloxetine for aromatase inhibitor-related arthralgia; and meditation, yoga, and exercise for QOL [32–36]. However, the effects of these supportive therapies have not been satisfactory, and approximately half of the patients discontinue ET at 5 years of treatment [16, 17, 37–40]. Recently, extended ET was offered even to patients with stage I breast cancer [41]. However, long-term ET raises concerns about further adverse events, including the incidence of endometrial cancer and pulmonary embolism with tamoxifen [42], osteoporosis, bone fracture, arthralgia, myalgia, and cardiovascular events with aromatase inhibitors [43, 44].

Interestingly, adjuvant ET also reduced IBTR (including local recurrence and second cancer) and contralateral breast cancer [14, 25, 45]. In addition, extended ET further reduced contralateral breast cancer [42, 46–48], equivalent to preventing the incidence of breast cancer [49]. Moreover, IBTR and contralateral breast cancer were more frequent than distant metastasis (6.9%, 5.2%, and 2.6% at 9 years after surgery). Therefore, clinicians should inform patients of the risk of developing a second breast cancer.

The patient involvement committee in JCOG was established in 2018. The committee requested the omission of excessive ET for breast cancer with an excellent prognosis because patients suffer from the side effects of long-term ET. This study determined the incidence of distant metastasis based on the risk of recurrence and the effectiveness of adjuvant ET but failed to conclude on a recommendation of adjuvant ET based on the predefined consensus. After several discussions in the committee, patients’ expected outcomes were diverse, with distant metastasis, survival, IBTR, and contralateral breast cancer all being important and prioritized by each individual. Therefore, the committee recommended that our findings should be used for shared decision-making for treatment selection rather than conducting a further prospective study.

### Limitations and strengths

The strengths of this study include its large patient cohort with long-term follow-up and reliable data based on individual medical records. However, this study suffered from biases associated with retrospective studies, such as inherent selection bias. In addition, mastectomy was one of the potential risk factors for distant metastasis. Previous large, randomized trials have demonstrated equal survival for mastectomy and breast-conserving therapy in early breast cancer [50–52]. However, a population-based study reported mastectomy as a poor prognostic factor because of the lack of an additional value of radiation therapy [53]. This study did not identify the cause of this finding. Therefore, the analysis was designed to measure associations between patient variables, treatments, and outcomes.

## Conclusions

The prognosis for patients with ER-positive and HER2-negative T1a/bN0M0 breast cancer was as favorable as that for genetically low-risk breast cancer and was stratified by clinical risks. Adjuvant ET improved the incidence of distant metastasis and overall survival with a small absolute risk difference. These findings support shared decision-making that considers the implementation of adjuvant ET by weighing the prevention of recurrence and second cancer, side effect, and QOL.

### Electronic supplementary material

Below is the link to the electronic supplementary material.

**Supplementary Material 1: Figure S1**. Study flow-chart

**Supplementary Material 2: Figure S2**. Cumulative incidence of distant metastasis and effect of endocrine therapy according to risk factors. Low NG and negative Ly (A), Low NG and positive Ly (B), high NG and negative Ly (C), and high grade and positive Ly (D). NG, nuclear grade; Ly, lymphatic invasion

**Supplementary Material 3: Figure S3**. Cumulative incidence of distant metastasis according to tumor size. T1a (A), T1b (B) tumors

**Supplementary Material 4: Table S1**. Risk of Overall Survival in Patients With T1a/b Breast Cancer. **Table S2**. Distant Metastasis According to Risk Classification. **Table S3**. Risk of Ipsilateral Breast Tumor Recurrence in Patients With T1a/b Breast Cancer.

## Data Availability

The data underlying this article cannot be directly shared due to the privacy of individuals that participated in the study. Access to these data can be provided to researchers under certain circumstances, pending approval by the Institutional Review Board of Nagoya City University and agreement with the JCOG Breast Cancer Study Group.
