# Supplementary figures and images for: Prognostic impact of adjuvant endocrine therapy for estrogen receptor-positive and HER2-negative T1a/bN0M0 breast cancer
Source: Breast Cancer Res Treat. 2023 Sep 9;202(3):473–83. doi: 10.1007/s10549-023-07097-6 (PMC10564809; doi:10.1007/s10549-023-07097-6)

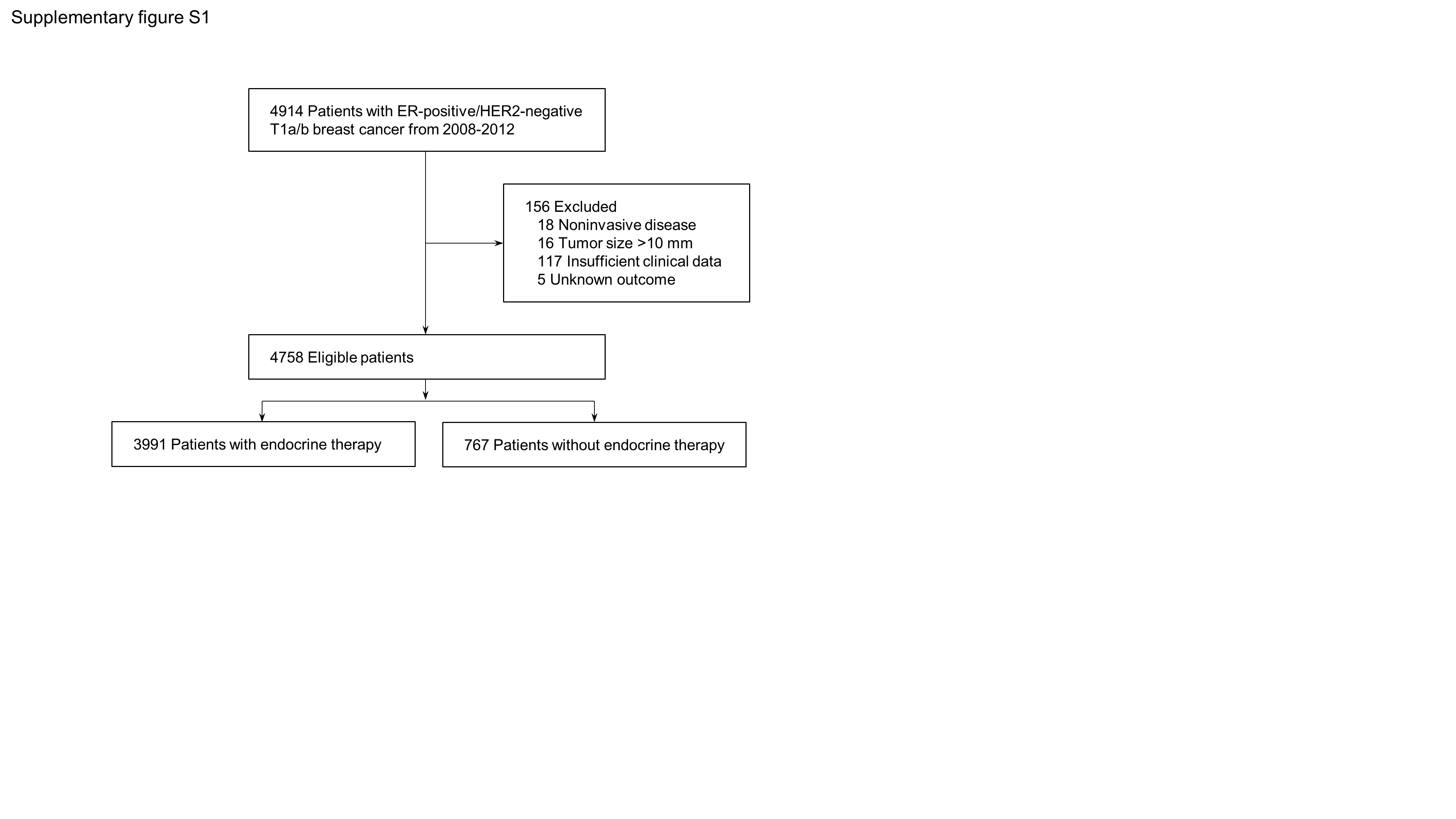

Supplement: Supplementary file 1 — Supplementary Material 1: Figure S1. Study flow-chart [file 10549_2023_7097_MOESM1_ESM.png]

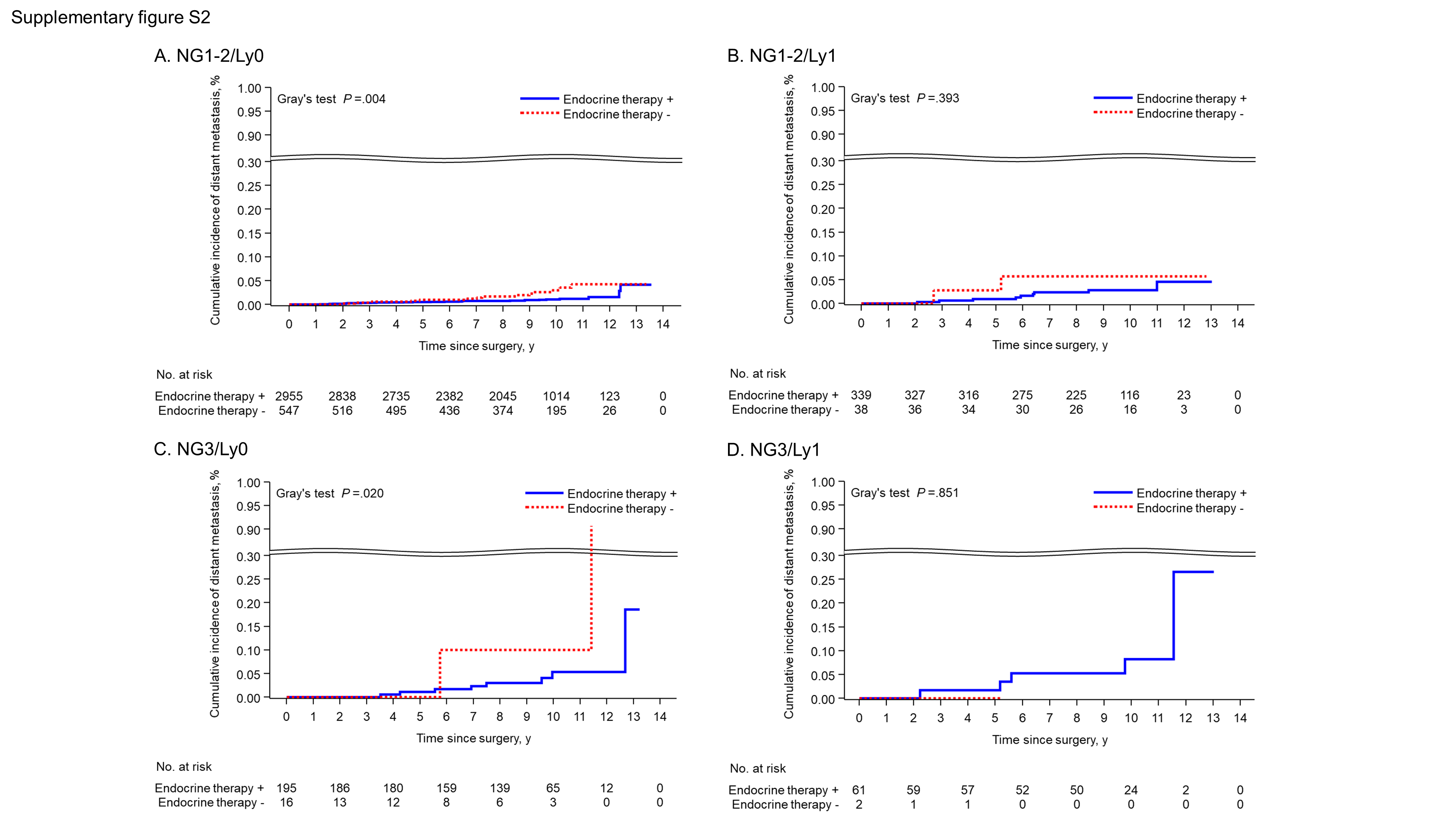

Supplement: Supplementary file 2 — Supplementary Material 2: Figure S2. Cumulative incidence of distant metastasis and effect of endocrine therapy according to risk factors. Low NG and negative Ly (A), Low NG and positive Ly (B), high NG and negative Ly (C), and high grade and positive Ly (D). NG, nuclear grade; Ly, lymphatic invasion [file 10549_2023_7097_MOESM2_ESM.png]

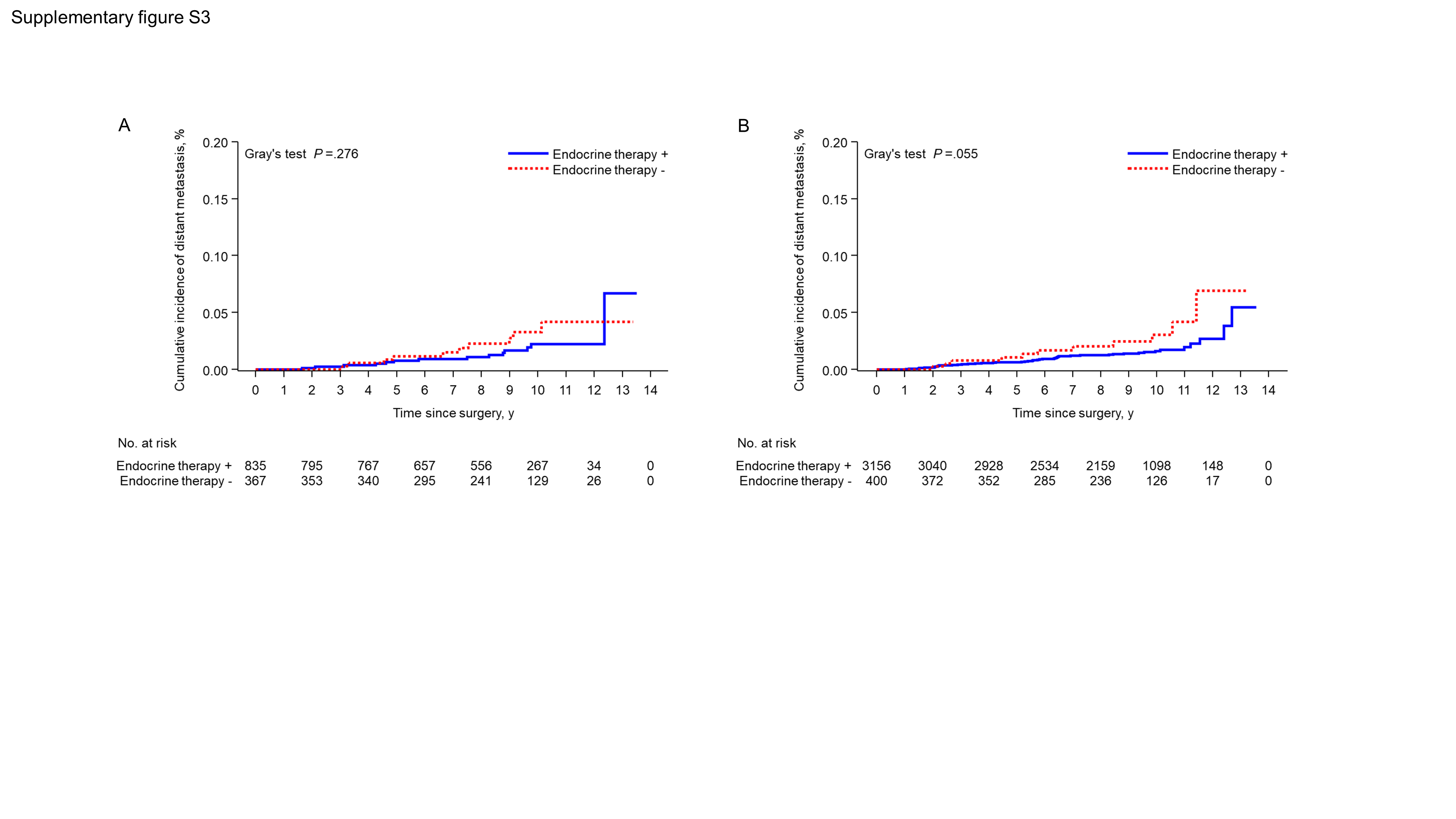

Supplement: Supplementary file 3 — Supplementary Material 3: Figure S3. Cumulative incidence of distant metastasis according to tumor size. T1a (A), T1b (B) tumors [file 10549_2023_7097_MOESM3_ESM.png]
