# Supplementary material for: Prognostic impact of adjuvant endocrine therapy for estrogen receptor-positive and HER2-negative T1a/bN0M0 breast cancer
Source: Breast Cancer Res Treat. 2023 Sep 9;202(3):473–83. doi: 10.1007/s10549-023-07097-6 (PMC10564809; doi:10.1007/s10549-023-07097-6)
Supplement: Supplementary file 4 — Supplementary Material 4: Table S1. Risk of Overall Survival in Patients With T1a/b Breast Cancer. Table S2. Distant Metastasis According to Risk Classification. Table S3. Risk of Ipsilateral Breast Tumor Recurrence in Patients With T1a/b Breast Cancer. [file 10549_2023_7097_MOESM4_ESM.docx]

**Supplementary table S1. Risk of Overall Survival in Patients With T1a/b Breast Cancer**

|  | Univariate analysis^a^ | | Multivariate analysis^a^ | |
| --- | --- | --- | --- | --- |
| Variable | HR (95% CI) | *P* value | HR (95% CI) | *P* value |
| Age, y |  |  |  |  |
| <55 | Reference |  | Reference |  |
| ≥55 | 2.44 (1.75–3.42) | <.001 | 2.51 (1.78–3.53) | <.001 |
| Tumor size, mm |  |  |  |  |
| ≤5 | Reference |  | Reference |  |
| >5 | 0.79 (0.57–1.11) | .177 | 0.84 (0.59–1.21) | .361 |
| Nuclear grade |  |  |  |  |
| 1 | Reference |  | Reference |  |
| 2 | 1.19 (0.82–1.72) | .352 | 1.09 (0.73–1.61) | .675 |
| 3 | 1.32 (0.73–2.41) | .360 | 1.42 (0.59–3.43) | .433 |
| Ki-67 labeling index, % |  |  |  |  |
| 0–9 | Reference |  | Reference |  |
| 10–19 | 1.32 (0.77–2.26) | .305 | 1.13 (0.73–1.77) | .584 |
| 20–29 | 1.12 (0.51–2.45) | .773 | 1.10 (0.57–2.14) | .772 |
| 30–39 | 0.59 (0.14–2.47) | .471 | 0.75 (0.22–2.52) | .644 |
| 40–100 | 1.11 (0.34–3.65) | .863 | 0.96 (0.35–2.65) | .942 |
| Lymphatic invasion | 1.56 (1.01–2.41) | .046 | 1.71 (1.09–2.70) | .020 |
| Vascular invasion | 0.49 (0.12–1.99) | .320 | 0.50 (0.12–2.03) | .334 |
| Endocrine therapy | 0.54 (0.38–0.77) | <.001 | 0.57 (0.39–0.83) | .004 |
| Surgical procedure |  |  |  |  |
| Mastectomy | Reference |  |  |  |
| Breast-conserving surgery | 0.82 (0.59–1.15) | .253 | 0.82 (0.58–1.16) | .260 |
| Radiation therapy | 0.69 (0.50–0.93) | .017 | 0.84 (0.54–1.31) | .440 |

Abbreviations: CI, confidence interval; NA, not applicable; HR, hazard ratio.

^a^ Estimated using the Cox proportional hazards models.

**Supplementary table S2. Distant Metastasis According to Risk Classification**

| Risk classification | Endocrine therapy | 9-year cumulative incidence of distant metastasis |
| --- | --- | --- |
| Nuclear grade 1–2  Lymphatic invasion negative | No | 2.3% (95%CI 1.1–4.0) |
|  | Yes | 0.9% (95%CI 0.6–1.3) |
| Nuclear grade 1–2  Lymphatic invasion positive | No | 5.7% (95%CI 1.0–17.0) |
|  | Yes | 2.8% (95%CI 1.3–5.3) |
| Nuclear grade 3  Lymphatic invasion negative | No | 10.0% (95%CI 0.5–37.4) |
|  | Yes | 3.0% (95%CI 1.1–6.5) |
| Nuclear grade 3  Lymphatic invasion positive | No | NA |
|  | Yes | 5.3% (95%CI 1.4–13.3) |

Abbreviations: CI, confidence interval; NA, not applicable.

**Supplementary table S3. Risk of Ipsilateral Breast Tumor Recurrence in Patients With T1a/b Breast Cancer**

|  | Univariate analysis^a^ | | Multivariate analysis^a^ | |
| --- | --- | --- | --- | --- |
| Variable | sHR (95% CI) | *P* value | sHR (95% CI) | *P* value |
| Age, y |  |  |  |  |
| <55 | Reference |  | Reference |  |
| ≥55 | 0.53 (0.32–0.87) | .012 | 0.46 (0.28–0.78) | .004 |
| Tumor size, mm |  |  |  |  |
| ≤5 | Reference |  | Reference |  |
| >5 | 0.65 (0.38–1.10) | .111 | 0.88 (0.49–1.58) | .677 |
| Nuclear grade |  |  |  |  |
| 1 | Reference |  | Reference |  |
| 2 | 1.95 (1.14–3.36) | .015 | 1.33 (0.76–2.34) | .315 |
| 3 | 1.98 (0.83–4.74) | .125 | 3.41 (0.99–11.74) | .052 |
| Ki-67 labeling index, % |  |  |  |  |
| 0–9 | Reference |  | Reference |  |
| 10–19 | 0.80 (0.38–1.65) | .541 | 0.85 (0.41–1.77) | .661 |
| 20–29 | 1.11 (0.42–2.93) | .840 | 0.86 (0.32–2.28) | .759 |
| 30–39 | 1.32 (0.39–4.47) | .650 | 0.86 (0.23–3.24) | .824 |
| 40–100 | NA | NA | 0.48 (0.10–2.25) | .352 |
| Lymphatic invasion | 1.46 (0.74–2.89) | .274 | 1.35 (0.67–2.73) | .401 |
| Vascular invasion | 2.24 (0.82–6.14) | .115 | 2.82 (1.01–7.88) | .048 |
| Endocrine therapy | 0.17 (0.11–0.28) | <.001 | 0.20 (0.12–0.34) | <.001 |
| Radiation therapy | 0.33 (0.20–0.54) | <.001 | 0.40 (0.23–0.68) | <.001 |

Abbreviations: CI, confidence interval; NA, not applicable; sHR, subdistribution hazard ratio.

^a^ Estimated using the Fine-Gray models.
